# Chitosan and its derivatives in Lady Rosetta potatoes:In vivo gene expression modulation driving growth, yield, quality, and antibacterial defense

**DOI:** 10.1038/s41598-026-48263-2

**Published:** 2026-05-25

**Authors:** Tasneem Yahya Zakaria, Mohamed El-Soda, Abdelhamid Fikry ElFoli, Mona H. Hussein, Ahmed M. M. Gabr

**Affiliations:** 1 Department of Genetics, Faculty of Agriculture, Cairo University, Giza, 12613 Egypt; 2 Organic Farming Consultant, Chitosan Egypt, Giza, Egypt; 3 Professor of Plant Biotechnology, Biotechnology Research Institute, National Research Centre (NRC), Cairo, 12622 Egypt

**Keywords:** Chitosan/Chitosan derivatives, Lady Rosetta, Minituber yield, *AS1* and *POT32* expression, Potato phytopathogens, Biostimulant/Bioprotectant, Biological techniques, Biotechnology, Microbiology, Plant sciences

## Abstract

This study examines how different forms and concentrations of chitosan affect the growth, minituber yield, and processing quality of the ‘Lady Rosetta’ potato variety. Using a two-way ANOVA method, we assessed the effects of chitosan, chitosan acetate, chitosan lactate, and N, O-Carboxymethyl chitosan, along with their interactions at various concentrations. The results show that the type of chitosan is the main factor for improvement. Specifically, chitosan lactate significantly accelerated germination to 4.1 days in Season 1 and 4.67 days in Season 2, while increasing shoot length to 37.8 cm and 35.5 cm, respectively—nearly double the height of the control group. Regarding physiological traits, a significant interaction between chitosan form and concentration was observed for all photosynthetic pigments. Notably, 0.01% chitosan acetate maximized chlorophyll *a* (28.8 mg/g), while 0.03% N, O-Carboxymethyl chitosan resulted in the highest carotenoid accumulation (8 mg/g). For yield, chitosan lactate achieved the highest results, reaching an average of 3.78 minitubers per plant and a weight of 21.33 g in Season 2, greatly surpassing other forms. Molecular analysis showed that chitosan lactate treatments significantly reduced *AS1* and *POT32* gene activity (up to 75% and 73% reduction, respectively), explaining the lower enzymatic browning and reduced acrylamide precursors. Additionally, 0.05% chitosan lactate showed strong antibacterial activity against *Pectobacterium carotovorum* and *Ralstonia solanacearum*, with inhibition zones reaching 24.66 mm. These results indicate that chitosan lactate serves as a dual-purpose biostimulant and bioprotectant, greatly enhancing both productivity and quality in potato farming.

## Introduction

The potato (*Solanum tuberosum L.*) has become one of the most widely farmed crops worldwide, noted for its adaptability in consumption as both a cooked vegetable and a fundamental component in several processed items, such as starch and chips. In Egypt, approximately 649,000 feddans are dedicated to potato cultivation^1^. Despite this substantial agricultural commitment, there remains a pressing need for the importation of virus-free minitubers to ensure healthy yields. Farmers often resort to the application of chemical fertilizers to optimize productivity; however, such practices can result in harmful chemical residues that pose significant health risks to consumers.

Considering these challenges, chitosan—a natural polysaccharide derived from chitin found in crustacean exoskeletons and fungal cell walls—has garnered attention as a viable organic alternative in agricultural practices^2,3^. Chitosan has demonstrated the ability to promote plant development, displaying biocompatibility, antibacterial and antifungal characteristics, and functioning as an efficient biodegradable chelating agent. Its zero-pre-harvest interval (PHI) and lack of residues make it an attractive option for integration into organic fertilizers, pesticides, and post-harvest applications.

Efforts to overcome the inherent limitations of chitosan have led to the development of its derivatives, such as chitosan acetate, chitosan lactate, and N, O-carboxymethyl chitosan (N, O-CMC), which exhibit improved solubility and enhanced antimicrobial properties^4,5^. These derivatives function as bio-stimulants, promoting crop productivity while reducing dependency on synthetic fertilizers^3^. Our previous in vitro study demonstrated that various forms and concentrations of chitosan and its derivatives—both regular and nano—significantly enhanced vegetative growth, chlorophyll content, and microtuber production in the Lady Rosetta potato cultivar, while also confirming genetic stability through RAPD and ISSR markers^6^.

The assessment of quality traits is essential for any effective crop improvement program, particularly for the potato industry, where parameters such as dry matter content, specific gravity, reducing sugars, starch content, chip color, and crispiness critically influence chip processing quality^7,8^. Variability exists among potato cultivars, with certain varieties exhibiting superior qualities suited for chip production^8,9^. Moreover, challenges such as enzymatic browning and acrylamide accumulation frequently compromise the quality of potato tubers, affecting food safety and nutritional value. Enzymatic browning, regulated by the *POT32* gene, accounts for approximately 50% of the losses in the industrial production of fruits and vegetables^10^. The formation of acrylamide—a neurotoxic and potentially carcinogenic compound—occurs during high-temperature cooking and is closely linked to the Maillard reaction, with asparagine as the limiting substrate^11,12^. Additionally, *Ralstonia solanacearum*, responsible for brown rot disease, and *Pectobacterium carotovorum*, which causes soft rot disease, are two primary pathogens that significantly impair the yield, quality, and storage longevity of potato crops^13–18^. This study aims to evaluate the effects of varying concentrations of chitosan and its derivatives on potato growth promotion, yield, quality traits, gene expression related to key physiological pathways, and resistance to prevalent diseases. This research seeks to contribute valuable insights into sustainable agricultural strategies for enhancing potato production while mitigating health risks associated with synthetic chemicals.

## Methods

The present in vivo study in potatoes (*Solanum tuberosum L*) was carried out at the Faculty of Agriculture, Cairo University, experiment station, Giza, Egypt. The experiment is repeated for two seasons. The details of materials used and experimental methods are as follows,

### Preparation of treatments and minitubers

Var. Lady Rosetta minitubers were kindly provided with the New Valley, Egypt Potato Research project. Minitubers were left at room temperature for five days before planting in pots.

**Fig. 1:**
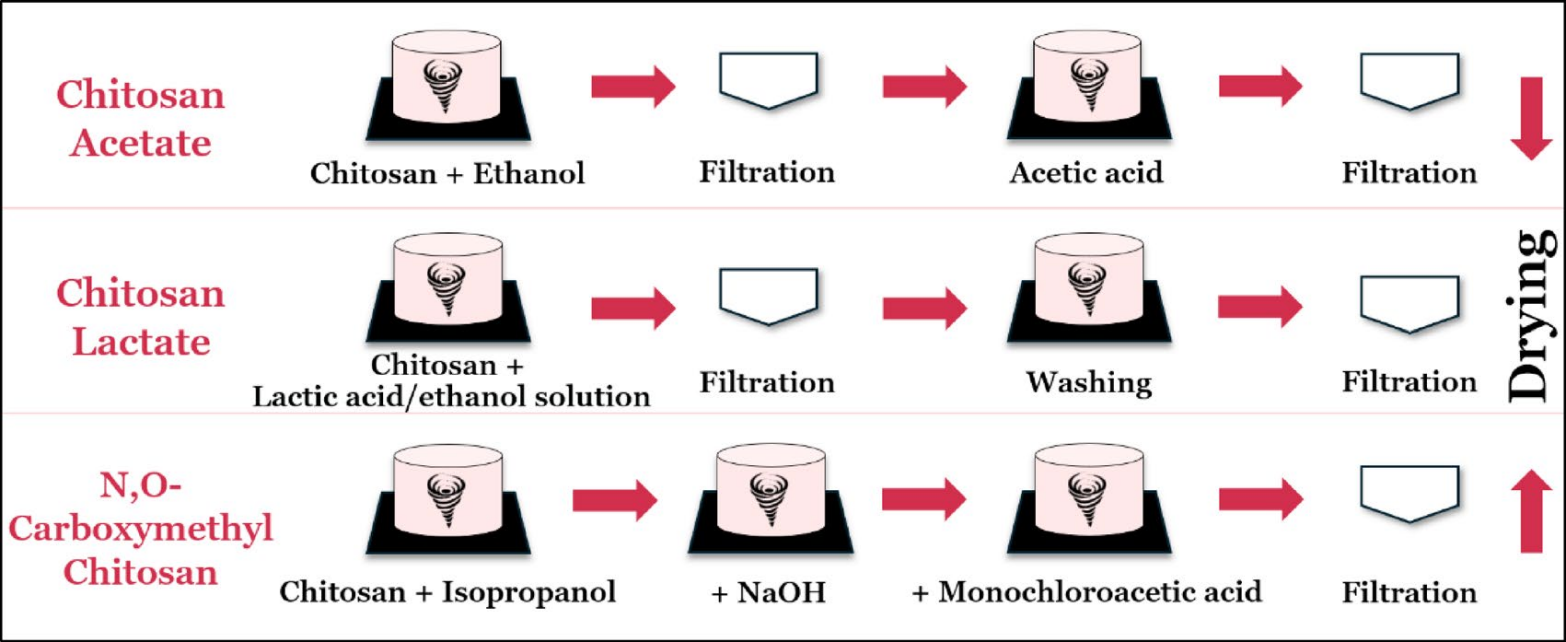
Chitosan derivatives (chitosan acetate, chitosan lactate, and N, O-CMC) preparation methods.

The chitosan used in this experimentation was of research-grade purity and was purchased from Chitosan Egypt. Chitosan derivatives were prepared according to the methods used by^19–21^ as shown in Fig. 1.

Every three tubers were soaked in a specific treatment for about 6–8 h directly before cultivation.

Different concentrations of chitosan and chitosan derivatives were used as treatments; T0 = control, T1 = 0.01% chitosan, T2 = 0.03% chitosan, T3 = 0.05% chitosan, T4 0.01% chitosan acetate, T5 = 0.03% chitosan acetate, T6 = 0.05% chitosan acetate, T7 = 0.01% chitosan lactate, T8 = 0.03% chitosan lactate, T9 = 0.05% chitosan lactate, T10 = 0.01% N, O-Carboxymethyl chitosan, T11 = 0.03% N, O-Carboxymethyl chitosan, T12 = 0.05% N, O-Carboxymethyl chitosan.

### Soil preparation

The soil used in the experiment was 1:1 peat moss: sand; it was mixed well and put in 40 cm diameter pots in an open-air area. The soil was irrigated with water 24 h before cultivation. Nitrogen, Phosphorus, and Potassium (NPK) (20:20:20) were used as a basic fertilizer for all pots during the experiment.

## Experimental design

Three replications and a fully randomized design (CRD) were used for the trials. Two-way Analysis of variance (ANOVA) was used to examine the data, using treatment as a source of variation. The Statistical Package for the Social Sciences (SPSS) software was used to do mean comparisons between the treatments using Tukey’s test at a 5% level of significance (*P ˂ 0.05*).

### Studying the effect of different treatments on vegetative growth for 2 seasons

The pots were half-filled with soil, and minitubers were cultivated one minituber per pot. Minitubers were irrigated regularly at 7-day intervals.

All pots were treated with their specific treatment (as a foliar treatment) at 30, 45, and 60 days after planting. Soil backfilling was done regularly to maximize the final tuber yield. Field conditions (temperature and humidity) were in the range of 27 °C ± 2 and 40% ±2, respectively.

### Studying the effect of different treatments on the leaf content of chlorophyll and carotenoids

Using^22^ method with minor modifications. Leaves were collected from promising treatment samples to measure their chlorophyll A, B, and carotenoid content. 0.1 g of leaves was added to 1 ml of acetone, the dark bottle underwent five minutes of ultrasonication at 50 °C, then the resulting liquid was centrifuged for five minutes at 10,000 rpm. Finally, the supernatant was used for measurements with a spectrophotometer; chlorophyll a, b, and carotenoids were measured at an absorbance of 645 nm, 662 nm, and 470 nm, respectively.

Chlorophyll a = 12.25 A_662_–2.79 A_645_

Chlorophyll b = 21.5 A_645_–5.1 A_662_

Carotenoids = (1,000 A_470_–1.82 chlorophyll a – 85.02 chlorophyll b) /198

### Studying the effect of different treatments on yield for 2 seasons

After 120 days of cultivation, the yield of both seasons is then harvested and kept for the upcoming analysis.

### Studying the effect of different treatments on the quality traits

The dry matter percentage was measured using the^23^ method, and the reducing sugar percentage was extracted using a method outlined in the^24^. Fat percentage was assessed according to^25^, while Specific gravity was measured using this formula Specific gravity = Weight of tuber in air/Weight of tuber in fresh water at 4 °C. Total Soluble Solids (TSS) were measured using a Hand Sugar Refractometer “ERMA” Japan, according to the^26^. Starch content was determined according to the method outlined in to^24^. Starch content was calculated using the glucose standard curve. Total sugars were also measured using the method outlined in^24^.

### Studying the effect of the promising treatments on gene expression for *AS1* and Polyphenol Oxidase (*PPO*) genes with real-time Polymerase Chain Reaction (PCR)

#### Extraction of Ribonucleic Acid (RNA)

In a 2 mL safe-lock tube, 100 mg of the sample was combined with 500 µl of plant RNA lysis solution and a tungsten carbide bead. After three minutes of incubation at 56 °C, the mixture was centrifuged for five minutes at 14,000 rpm. The supernatant was supplemented with 250 µL of 96% ethanol. After that, the mixture was sent to a purification column. After that, the lysate was cleaned and centrifuged per the manufacturer’s instructions. 50 µl of the elution buffer included in the kit was used to elute the nucleic acid. To get rid of any remaining Deoxyribonucleic Acid (DNA), DNase digestion was used.

### Real-Time PCR conditions and primer design

#### PCR reaction setup

The following ingredients were used to create each 25 µl SYBR Green real-time PCR reaction: 0.5 µl of each of the forward and reverse primers (20 pmol), 8.25 µl of RNase-free water, 3 µl of RNA template, 0.25 µl of reverse transcriptase, and 12.5 µl of 2× QuantiTect SYBR Green PCR Master Mix (Qiagen).

#### Primer design

Gene-specific primers were designed and synthesized for Asparagine Synthetase 1 (*AS1*) and Polyphenol Oxidase (*POT32*), while *Tubulin1* primers were adapted from^27^ and used as the housekeeping gene. The sequences are as follows:

Tubulin1 forward primer: GTCAGTCTGGTGCTGGTAATAA and reverse: TCTCAGCCTCCTTCCTTACA, Asparagine Synthetase 1 (*AS1*).

Forward primer: AAGGAGGAGTTCCACAGGGA, and reverse: GTCGGAGTGTTGTGTGGGAA, Polyphenol Oxidase (*POT32*).

Forward primer: ACGTGACCAAAGTCACCGAA, and reverse primer: GGCGGAAGTGAAGCTGTATT.

#### Thermal cycling conditions

Reverse transcription was conducted at 50 °C for 30 min, followed by an initial denaturation at 94 °C for 15 min. PCR amplification was carried out for 40 cycles, consisting of denaturation at 94 °C for 15 s, annealing at gene-specific temperatures (Tubulin1: 60 °C; *AS1*: 57 °C; *POT32*: 55 °C) for 30 s, and extension at 72 °C for 40 s.

Dissociation curve analysis was performed at the end of amplification to verify the specificity of each reaction. This included secondary denaturation at 94 °C for 1 min, annealing at the respective annealing temperature for 1 min, and a final denaturation step at 94 °C for 1 min.

### Assessment of the bactericidal effect of promising treatments

**Fig. 2:**
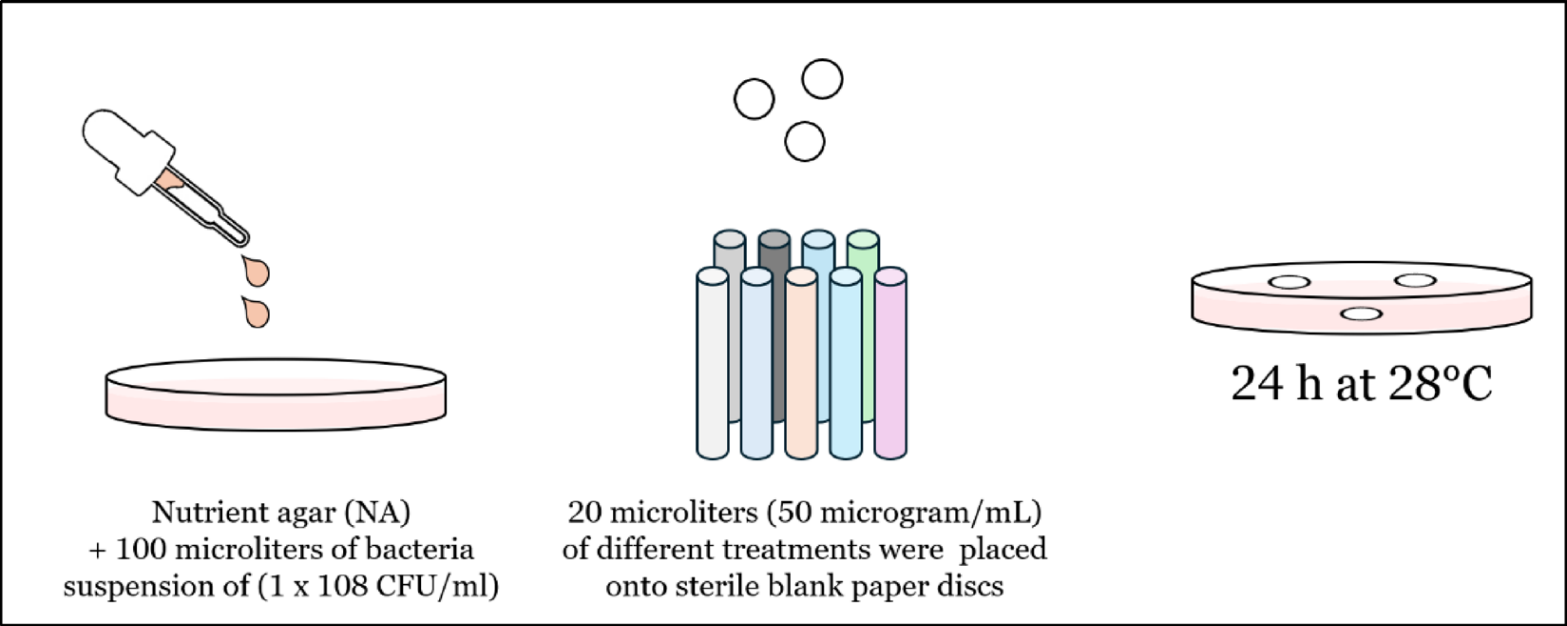
Antibacterial assessment methodology.

Figure 2, shows how *Pectobacterium carotovorum* and *Ralstonia solanacearum* were used in antibacterial experiments utilizing the methods of^28^. They were grown fresh in Luria-Bertani medium (LB) and treated with 100 µl of bacterial suspension containing 1 × 108 Colony-Forming Unit (CFU)/mL on the surface of nutritional agar (NA) (Quelab, Montreal, Canada, 22 g/L). After forty-five minutes, twenty microliters of each treatment were placed onto sterile blank paper discs at a final concentration of fifty micrograms per milliliter. Each of the experiments was conducted in three replicates. The widths of the bacterial growth inhibition zones surrounding the discs were measured following a 24-hour incubation period at 28°C. The positive control was novobiocin (750 mg), whereas the negative control was sterilized water. Following the incubation time, the diameter of the growth inhibition zones was assessed.

## Results

### Effect of chitosan forms and concentrations on vegetative growth (S1 and S2)

#### Statistical overview

As detailed in Table 1, the two-way ANOVA indicated that the **chitosan form (F)** was the main influence on growth, having a highly significant effect (*P* < 0.05) on all growth parameters except for branch number in Season (1) Conversely, the **concentration (C)** did not have a significant effect on growth parameters (*P* > 0.05). A **notable interaction (F x C)** was found for leaf number in both seasons, as well as for germination and shoot length in Season (2) This suggests that for these traits, the effectiveness of the chitosan form was affected by the applied concentration.

### Germination and shoot length

In both seasons, **chitosan lactate** consistently outperformed the control and all other forms, leading to the fastest germination times (4.1 days in S1 and 4.67 days in S2) (Table 1). It also produced the tallest plants, reaching 37.8 cm in S1 and 35.56 cm in S2—almost double the height of the control group (19.33 cm and 22.67 cm). Although **chitosan acetate and N**,** O-CMC** showed intermediate performance, the statistical significance of the form factor (*P* = 0.046 and *P* < 0.001) highlights its important role in shoot growth.

### Number of shoots and branches

The number of shoots was significantly affected by the chitosan form in both seasons (*P* = 0.001 and *P* < 0.001). **Chitosan lactate** produced the highest density of shoots (3.56 in S1 and 3.33 in S2), outperforming the control (Table 1). For branching, there were no significant differences in Season 1 (*P* = 0.269). However, in Season 2, both **lactate and acetate** forms significantly increased branch counts compared to the control group.

### Number of leaves per shoot

A significant **interaction (F x C)** was found for leaf count in both Season 1 (*P* = 0.002) and Season 2 (*P* = 0.000). **Chitosan lactate** generally had the highest leaf counts (reaching 27.25 in S1), but the interaction indicates that the best leafing response comes from specific combinations of chitosan form and concentration (Table 1).

**Table 1:** Main effects and interaction of different chitosan forms and concentrations on the germination and vegetative growth parameters of potato minitubers during two consecutive growing seasons (S1 and S2).

Treatment Factor	Level	Days to germinate / plant		Shoot length (cm) / plant		Number of shoots / plant		Number of branches / plant		Number of leaves/shoots/ plant	
		S1	S2	S1	S2	S1	S2	S1	S2	S1	S2
**Control**	T0 (None)	8^a^	8.3^a^	19.3^a^	22.6^a^	1.3^a^	1.3^a^	5^a^	8.6^a^	13.9^a^	14^a^
Chitosan Form (F)	Chitosan	7.5^a^	10.2^b^	28^ab^	30.2^b^	2.1^ab^	1.8^ab^	11.6^b^	9.4^a^	16.8^ab^	16.1^ab^
	Acetate	6.5^ab^	7.5^ab^	32.3^b^	28.8^b^	2^ab^	2.22^b^	12.4^b^	11^ab^	21.5^b^	18.3^b^
	**Lactate**	**4.1** ^b^	**4.67** ^c^	**37.8** ^c^	**35.5** ^c^	**3.5** ^c^	**3.3** ^c^	**15.3** ^c^	**13.4** ^b^	**27.2** ^c^	**17** ^ab^
	N, O-CMC	7.1^a^	7.3^ab^	29.4^ab^	30.3^b^	2.3^ab^	1.7^a^	11.8^b^	11^ab^	16.9^ab^	14.2^a^
Concentration (C)	0.01%	6	8.2	31.1	31.8	2.4	2	13.1	10.9	21.8	16.7
	0.03%	6.3	7	35.3	32.6	2.5	2.2	13	11.2	19.1	16.6
	0.05%	6.6	7	29.2	29.25	2.50	2.58	12.25	11.50	20.96	15.83
ANOVA (*P*-value)	Form (F)	**0.006**	**< 0.001**	**0.046**	**< 0.001**	**0.001**	**< 0.001**	0.269	**< 0.001**	**< 0.001**	**< 0.001**
	Conc (C)	0.725	0.33	0.147	0.13	0.88	0.32	0.84	0.64	0.32	0.32
	**F x C Interaction**	0.89	**0.040**	0.23	**0.022**	0.19	0.33	0.60	0.32	**0.002**	**0.000**

Values are presented as means. Means within a column followed by the same lowercase letter are not significantly different at the 5% probability level (*P* < 0.05) based on Tukey’s Honestly Significant Difference (HSD) post-hoc test. Bolded values in the ANOVA section indicate significant main effects or interactions. Bolded values in the treatment rows indicate the highest performing treatment for that parameter.

### Effect of different treatments on the leaf content of Photosynthetic Pigments Content

The accumulation of photosynthetic pigments was significantly influenced by both the chemical form and application concentration of chitosan. **Chlorophyll** ***a*** synthesis was most effectively promoted by **0.01% chitosan acetate** (T4), which achieved a peak concentration of 28.8 mg/g, significantly exceeding the control and other treatment groups (Table 2). Similarly, **chlorophyll** ***b*** levels were maximized in plants treated with **0.01% chitosan acetate** (T1; 28.8 mg/g) and **0.01% chitosan lactate** (T7; 28.5 mg/g).

**Carotenoid** content exhibited a distinct response pattern across treatment combinations. The highest carotenoid accumulation was observed under **0.03% N, O-CMC** (T11; 8 mg/g), followed by **0.05% chitosan** (T3; 7.2 mg/g) and **0.01% chitosan acetate** (T4; 6.9 mg/g). These data suggest a specialized physiological response: while lower concentrations of acetate and lactate derivatives specifically enhance chlorophyll biosynthesis, the carboxymethyl form at moderate concentrations is superior for carotenoid enrichment.

**Table 2:** Two-way ANOVA summary of the effects of Chitosan Form (F), Concentration (C), and their Interaction (F × C) on vegetative growth and photosynthetic pigments.

Source of Variation	Chlorophyll a	Chlorophyll b	Carotenoids
Chitosan Form (F)	*P* < 0.05*	*P* < 0.05*	*P* < 0.01**
Concentration (C)	*P* < 0.05*	*P* < 0.05*	*P* < 0.05*
Interaction (F × C)	*P* < 0.05*	*P* < 0.05*	*P* < 0.05*

*Significant at *P* < 0.05; ** Highly significant at *P* < 0.01.

### Impact of chitosan forms and concentrations on Minituber Yield

#### Statistical Summary

The two-way ANOVA results for minituber yield are shown in Table 3. The **chitosan form (F)** significantly affected the number of minitubers in both Season 1 (*P* = 0.021) and Season 2 (*P* = 0.003), as well as the weight of minitubers in Season 2 (*P* = 0.013). While the **concentration (C)** alone did not have a significant effect (*P* > 0.05), a significant **interaction (F x C)** was found for minituber weight in both Season 1 (*P* = 0.018) and Season 2 (*P* = 0.001). This suggests that the effect of chitosan on tuber weight is closely tied to the specific concentration used.

### Number of minitubers

In Season 1, all chitosan forms generally increased minituber counts over the control, with **chitosan lactate and N**,** O-CMC** showing the highest average counts (4.44 and 4.11, respectively). By Season 2, **chitosan lactate** significantly surpassed the others with an average of 3.78 minitubers per plant, compared to just 1.33 in the control. **Acetate and N**,** O-CMC** had intermediate results, while the **standard chitosan** form remained statistically similar to the control.

### Weight of minitubers

The weight of minitubers was significantly increased by the treatment combinations, especially in the second season. In Season 2, **chitosan lactate** achieved the highest average weight (21.33 g), followed by **N**,** O-CMC** (18.56 g), both significantly higher than the control (13.67 g). The significant **interaction (F x C)** in both seasons (*P* = 0.018 and *P* = 0.001) highlights that the **lactate** form, particularly at specific concentrations, offers the most potential for increasing tuber weight.

**Table 3:** Impact of chitosan chemical forms, application concentrations, and their interaction on the number and weight of potato minitubers produced in Season 1 and Season 2.

Treatment Factor	Level	Number of Minitubers/plant		Weight of Minitubers (g)/plant	
		S1	S2	S1	S2
Control	T0 (None)	3.67^a^	1.33^a^	10.67^a^	13.67^a^
Chitosan Form (F)	Chitosan	2^a^	2^a^	12.22^a^	17^ab^
	Acetate	1.78^a^	2.22^ab^	19.11^a^	17^ab^
	**Lactate**	**4.44** ^**a**^	**3.78** ^**b**^	**19.11** ^**a**^	**21.33** ^**b**^
	N, O-CMC	4.11^a^	2.44^ab^	18^a^	18.56^b^
Concentration (C)	0.01%	3.17	2.42	17.83	18.67
	0.03%	2.67	3	14.83	18.58
	0.05%	3.42	2.42	18.67	18.17
ANOVA (*P*-value)	Form (F)	**0.021**	**0.003**	0.102	**0.013**
	Conc (C)	0.682	0.247	0.336	0.906
	**F × C Interaction**	0.557	0.056	**0.018**	**0.001**

Values are presented as means. Means within a column followed by the same lowercase letter are not significantly different at the 5% probability level (*P* < 0.05) based on Tukey’s Honestly Significant Difference (HSD) post-hoc test. Bolded values in the ANOVA section indicate significant main effects or interactions. Bolded values in the treatment rows indicate the highest performing treatment for that parameter.

### Effect of chitosan forms and concentrations on tuber quality traits

The most effective combinations—specifically **chitosan** (0.01%), **chitosan acetate** (0.03%), **chitosan lactate** (0.01%, 0.03%, and 0.05%), and **N**,** O-CMC** (0.01%)—were tested against the control for various quality measures.

### Dry matter and fat content

**Fig. 3:**
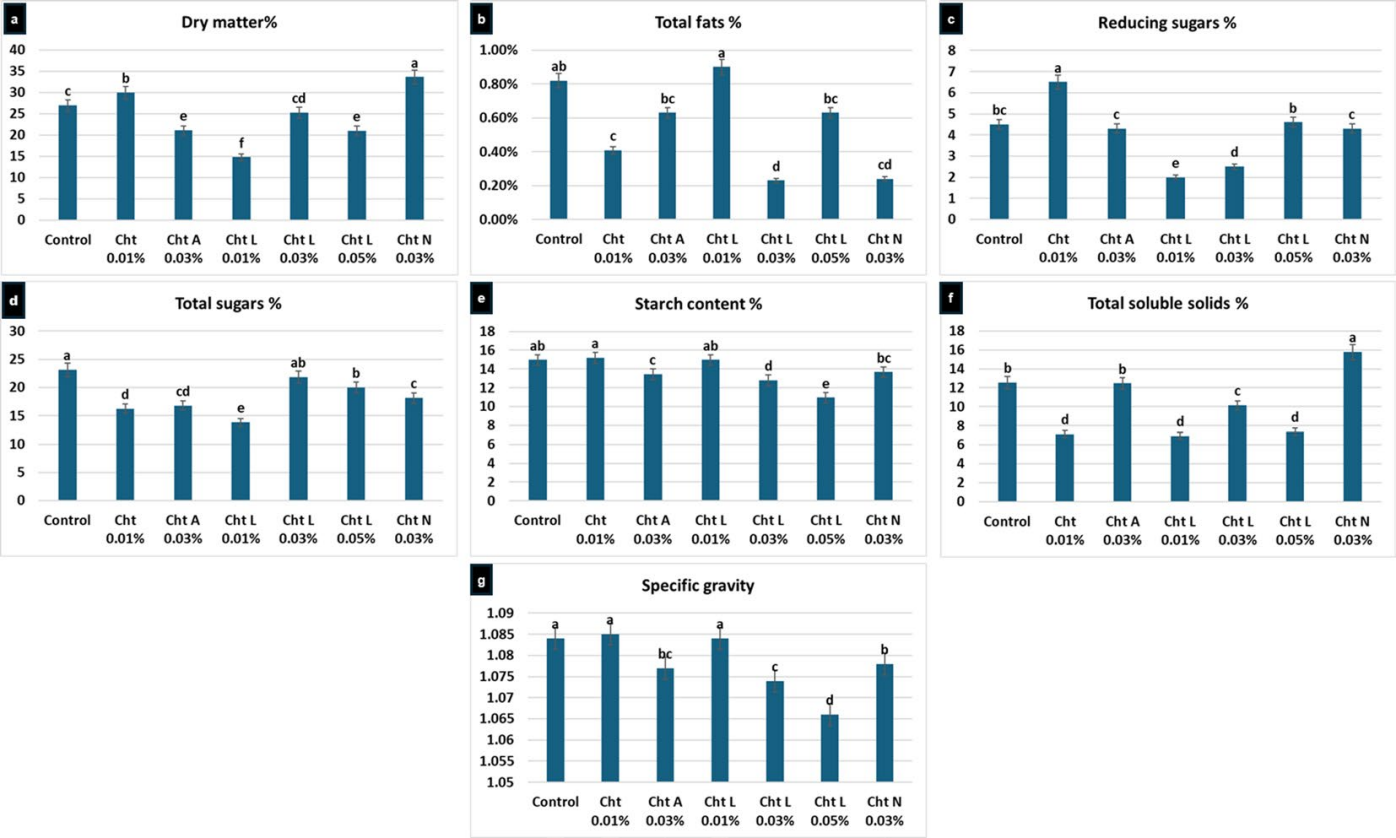
Average effect of treatments on quality traits: dry matter, total fats, reducing sugars, total sugars, starch content, total soluble solids, and specific gravity Values followed by different lowercase letters are significantly different according to Tukey’s Honestly Significant Difference (HSD) test at *P < 0.05*. Error bars reflect the standard deviation (SD) of the mean (*n* = 3).

The evaluation of tuber composition showed significant differences among treatments. The highest dry matter percentages were found with **0.03% N**,** O-CMC** (33.6%) and **0.01% chitosan** (29.9%) (Fig. 3a). Concerning fat content (Fig. 3b), the lowest levels were observed in **0.03% chitosan lactate** (0.23%) and **0.03% N**,** O-CMC** (0.24%), indicating these treatments might change how storage compounds are allocated.

### Sugar and starch profiles

The carbohydrate content was notably affected by the type of chitosan. Treatments with **0.01% and 0.03% chitosan lactate** resulted in the lowest reducing sugar levels (2% and 2.5%, respectively; Fig. 3c**)**. Similarly, **0.01% chitosan lactate** had the lowest total sugar content (13.8%), followed by **0.01% chitosan** (16.25%) and **0.03% chitosan acetate** (16.8%), showing a significant reduction from other treatments (Fig. 3d). In contrast, starch content analysis (Fig. 3e) showed that the control, **0.01% chitosan**, and **0.01% chitosan lactate** had the highest levels (about 15.0% to 15.15%).

### TSS and specific gravity

Total soluble solids (TSS) were significantly impacted by the treatment type (Fig. 3f). The lowest TSS was measured in tubers treated with **0.01% chitosan lactate** (6.93%), followed by **0.01% chitosan** (7.13%) and **0.05% chitosan lactate** (7.37%). However, specific gravity analysis (Fig. 3g) found no significant differences (*P* > 0.05) between the control and any chitosan treatments, indicating that, while internal chemical makeup changed, the physical density of the tubers did not vary throughout the study.

**Fig. 4:**
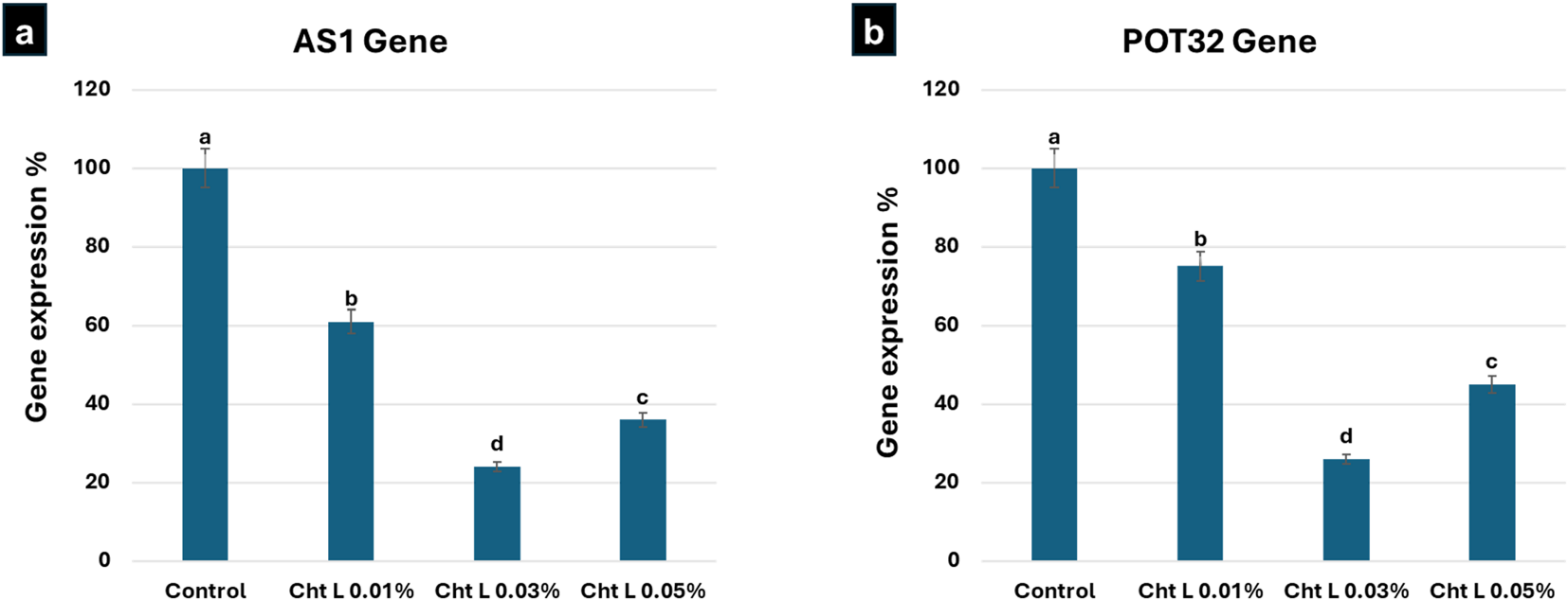
ِِAverage effect of treatments on *AS1* and *POT32* genes’ expression. Different letters indicate significant differences between treatments at *P < 0.05*. Error bars reflect the standard deviation (SD) of the mean (*n* = 3).

### Effect of Chitosan Lactate on Relative mRNA Expression of *AS1* and *POT32* Genes

We analyzed the relative mRNA expression of ***AS1*** and ***POT32*** genes to assess the potential of chitosan treatments in improving quality and health in the ‘Lady Rosetta’ variety. Expression levels were measured using real-time PCR (RT-PCR), with ***Tubulin 1*** as the control gene for normalization. The amplification curves for *Tubulin 1*, *AS1*, and *POT32* confirmed the study’s focus on gene expression.

### Relative Expression of the *AS1* Gene

Chitosan lactate significantly reduced the mRNA expression of the ***AS1*** gene compared to the control group (Fig. 4a). Among the tested concentrations, **0.03% chitosan lactate** (T8) was the most effective, resulting in a 75% reduction in expression. This was followed by **0.05% chitosan lactate** (T9) and **0.01% chitosan lactate** (T7), which caused reductions of 63% and 38%, respectively. All three lactate concentrations showed significant differences from the untreated control (T0).

### Relative Expression of the *POT32* Gene

Similar inhibitory patterns were observed for the ***POT32*** (*PPO*) gene expression (Fig. 4b). **Chitosan lactate at 0.03%** (T8) produced the largest decrease, lowering mRNA expression by 73% compared to the control. The treatments with **0.05% chitosan lactate** (T9) and **0.01% chitosan lactate** (T7) followed, with reductions of 54.6% and 24.5%. These results indicate significant, concentration-based suppression of the ***POT32*** gene by the lactate form, which differed from the control.

### Assessment of the bactericidal effect of chitosan and its derivatives

The antibacterial activity of selected chitosan treatments was tested against two key potato pathogens, *Pectobacterium carotovorum* and *Ralstonia solanacearum*, by measuring the size of the growth inhibition zones. The negative control, made of sterilized water, showed no antibacterial activity. **Novobiocin** (750 mg) served as a positive control antibiotic for comparison.

### Inhibition of *Pectobacterium carotovorum*

**Fig. 5:**
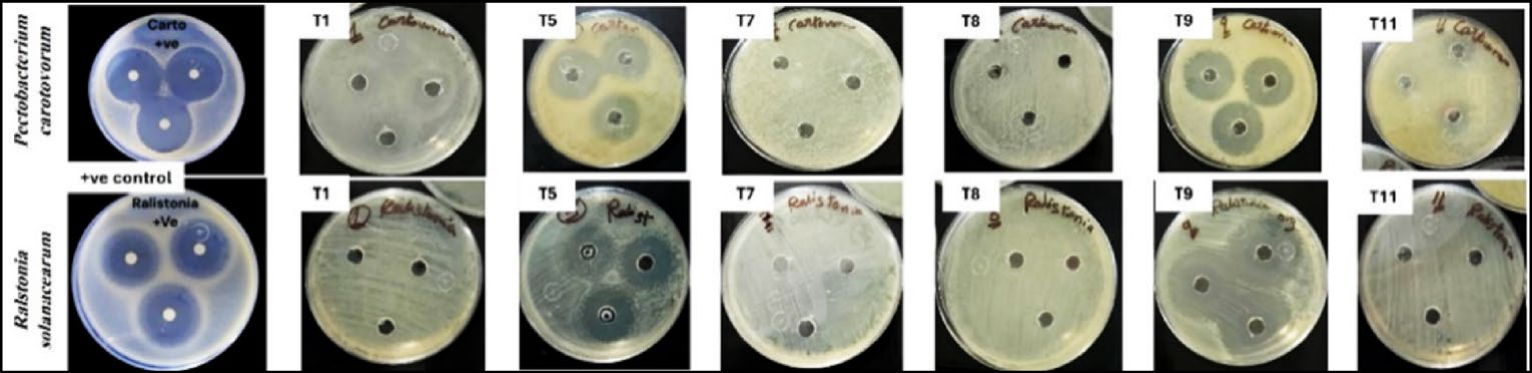
Inhibition zones for both *Pectobacterium carotovorum* and *Ralstonia solanacearum*.

The tested chitosan derivatives showed different levels of inhibitory effects against *P. carotovorum* **(**Fig. 5**)**. The most effective treatment was **0.05% chitosan lactate** (T9), which created an inhibition zone of **19 mm**. It was followed closely by **0.03% chitosan acetate** (T5) and **0.03% N, O-CMC** (T11), which produced inhibition zones of **18.66 mm** and **15 mm**, respectively.

### Inhibition of  *Ralstonia solanacearum*

For *R. solanacearum*, **0.05% chitosan lactate** (T9) showed the best antibacterial effect, with the largest inhibition zone measuring **24.66 mm (**Fig. 5**)**. Furthermore, **0.03% chitosan acetate** (T5) demonstrated a notable inhibitory effect with a zone diameter of **15.33 mm**. These results show that the lactate form at its highest concentration (0.05%) has the strongest bactericidal properties against both tested pathogens.

The results indicate that the lactate form at its highest concentration (0.05%) has the strongest bactericidal properties against both tested pathogens. Notably, the strong antibacterial activity seen with 0.05% chitosan lactate (T9) links to the increased plant growth and higher minituber yield found in the greenhouse trials, suggesting that this derivative offers both growth support and protective benefits.

## Discussion

The use of chitosan and its derivatives has shown promise for improving growth and quality in the ‘Lady Rosetta’ potato variety. Our Two-way ANOVA results underscore that while both chemical form and concentration are influential, their significant interaction (F x C) is the primary driver behind the physiological shifts observed (Tables 2 & 3). This suggests that the efficacy of chitosan is not merely dose-dependent but is fundamentally governed by the structural modifications of the polymer. This finding supports previous research, which highlighted the benefits of chitosan on plant growth^29^.

### Growth promotion and bio-protection synergy

The notable increase in germination rates and shoot elongation observed with Chitosan Lactate, specifically at 0.05% (T9), indicates a strong biostimulatory effect. This quick development may result from changes in seed physiology and better nutrient movement^29^. The critical analysis of our greenhouse trials reveals a strong correlation between the antibacterial efficacy (24.66 mm inhibition against *R. solanacearum*) and vegetative vigor. By significantly reducing the pathogen load, Chitosan Lactate acts as a metabolic shield, allowing the plant to reallocate energy from defense-related pathways toward primary growth and minituber initiation. Importantly, the strong antibacterial effect seen with 0.05% chitosan lactate (24.66 mm inhibition against *R. solanacearum*) directly connects to the increased vegetative growth and higher minituber yield seen in our greenhouse trials. This protection likely lowered the pathogen load and biotic stress on the minitubers during germination, allowing the plants to use more energy for growth instead of defense. This dual role, as both a biostimulant and a bioprotectant, clearly shows the effectiveness of the lactate form in a controlled greenhouse setting.

### Physiological and photosynthetic enhancements

The significant improvements in chlorophyll and carotenoid levels across treatments are crucial for effective photosynthesis. Our results further indicate that these enhancements are governed by a significant interaction between the chemical form and its concentration (Table 2), suggesting that the biostimulatory impact on the photosynthetic apparatus is highly specific to the derivative used. These enhancements driven by the F x C interaction (Table 2), directly fuel the "source-to-sink" relationship, as evidenced by the significant increase in minituber yield (Table 3).  This targeted enhancement of the photosynthetic apparatus is supported by^30^. Chitosan derivatives, especially chitosan lactate and N, O-CMC, seem to influence nitrogen transport and uptake in leaves^31^. Chitosan reportedly improves nitrogen movement by affecting transporters and metabolic pathways while also increasing antioxidant capacity and osmolyte levels^31^. This helps reduce oxidative stress and maintain cell function under study conditions^29^. These combined effects ensure efficient nitrogen allocation to growing tissues, supporting strong growth and more tubers per plant, consistent with our earlier in vitro research^32^.

### Molecular basis for tuber quality and processing safety

In terms of quality traits, 0.03% N, O-CMC and 0.01% chitosan achieved the highest dry matter percentages, which are important for making crisp chips with lower oil absorption^33^. However, the most notable results involve lowering reducing sugars and changing gene expression. A key highlight of this study is the transcriptional silencing of the *AS1* and *POT32 *genes. The Tukey HSD analysis confirms that Chitosan Lactate 0.03% achieved a significant down-regulation, reducing *AS1 *expression by 75% and *POT32 *by 73%. The suppression of *POT32 *(*PPO*) directly explains the reduction in enzymatic browning, while the silencing of *AS1 *is a crucial safety milestone. By reducing asparagine precursors at the molecular level, we effectively mitigate the risk of acrylamide formation—a known carcinogen—during high-temperature frying. This molecular shift, combined with the significant reduction in reducing sugars, confirms that lactate derivatives can bio-engineer a tuber that meets the most stringent industrial quality standards. This enhances the suitability and safety of the ‘Lady Rosetta’ variety for industrial use.

### Implications for sustainable agriculture and future research

From an agronomic perspective, the use of Chitosan Lactate and N, O-CMC offers a high-value, eco-friendly alternative to synthetic growth regulators and pesticides. The significant increase in Dry Matter % and Specific Gravity (Figure 3) translates to higher chip yield and lower oil absorption during processing, providing a direct economic benefit to the potato industry. Safety & Sustainability: By integrating these derivatives, growers can produce "processing-ready" minitubers that are safer for human consumption due to lower acrylamide potential. Despite these encouraging findings, several gaps in knowledge remain. The significant interaction observed for leaf number and germination shows that the effectiveness of chitosan isn’t universal; it depends on optimizing both its chemical form and concentration. While this study establishes a solid foundation for the ‘Lady Rosetta’ variety, future research should conduct larger field trials across various cultivars to evaluate long-term effects on soil health and microbial communities. Creating standardized methods for producing chitosan lactate and N, O-CMC is crucial for achieving dependable agricultural results. This indicates that a "one-size-fits-all" approach is insufficient, and future strategies must involve precision application based on specific cultivar requirements.

## Conclusion

In summary, the in vivo results indicate that chitosan lactate, especially at concentrations between 0.03% and 0.05%, significantly improves the growth, yield, and quality of potato minitubers. These findings are consistent with our earlier in vitro studies^32^, confirming that the lactate form enhances the solubility and effectiveness of chitosan. By reducing pathogenic bacteria and silencing genes linked to browning and acrylamide precursors, these derivatives could play an important role in sustainable potato production.

## Data Availability

The datasets generated during and/or analyzed during the current study are available from the corresponding author on reasonable request.
